# Metabolic Biomarkers Differentiate a Surgical Intervertebral Disc from a Nonsurgical Intervertebral Disc

**DOI:** 10.3390/ijms241310572

**Published:** 2023-06-24

**Authors:** Beata Toczylowska, Michal Woznica, Elzbieta Zieminska, Leszek Krolicki

**Affiliations:** 1 Nalecz Institute of Biocybernetics and Biomedical Engineering, Polish Academy of Sciences, 02-109 Warsaw, Poland; beata.toczylowska@ibib.waw.pl; 2 Spinal Unit, 7th Navy Hospital, 80-305 Gdansk, Poland; woznim@wp.pl; 3 Mossakowski Medical Research Institute, Polish Academy of Sciences, 02-106 Warsaw, Poland; 4 Department of Nuclear Medicine, Warsaw Medical University, 02-091 Warsaw, Poland; leszek.krolicki@wum.edu.pl

**Keywords:** nucleus pulposus, metabolic profiles, NMR spectroscopy, dehydrated intervertebral disc

## Abstract

Background: Degeneration of the intervertebral disc (IVD) is caused by disturbances in metabolic processes, which lead to structural disorders. The aim of this report is to analyze metabolic disorders in the degeneration process by comparing control discs with degenerated discs. In our research on the nucleus pulposus (NP), we used NMR spectroscopy of extracts of hydrophilic and hydrophobic compounds of the tissue. Methods: Nuclear magnetic resonance (NMR) spectroscopy allows the study of biochemistry and cellular metabolism in vitro. Hydrophilic and hydrophobic compounds were extracted from the NP of the intervertebral disc. In the NMR spectra, metabolites were identified and quantitatively analyzed. The results of our research indicate disturbances in the biosynthesis and metabolism of cholesterol, the biosynthesis and degradation of various fatty acid groups, ketone bodies, or lysine, and the metabolism of glycerophospholipids, purines, glycine, inositol, galactose, alanine, glutamate, and pyruvate in the biosynthesis of valine and isoleucine, leucine. All these disorders indicate pathomechanisms related to oxidative stress, energy, neurotransmission disturbances, and disturbances in the structure and functioning of cell membranes, inflammation, or chronic pain generators. Conclusions: NMR spectroscopy allows the identification of metabolites differentiating surgical from nonsurgical discs. These data may provide guidance in in vivo MRS studies in assessing the severity of lesions of the disc.

## 1. Introduction

Degeneration of the intervertebral disc (IVD) and the associated chronic lower back pain are serious health and economic problems. The costs of treating patients are very high. At the same time, there are no effective therapies to counteract degenerative changes and their effects. The disease is caused by disturbances in metabolic processes, which lead to structural disorders. The intervertebral disc consists of a gel-like nucleus pulpous (NP) and a surrounding fibrous ring (AF). The NP of a healthy adult disc is low in cells and rich in proteoglycans. As a result, the NP contains a large amount of water and thus fulfills its mechanical function. Resident cells are responsible for the biochemical composition of the NP through the production of proteoglycans, mainly aggrecan and collagen. IVD degeneration is characterized by an early decrease in cellularity of the NP region and associated extracellular matrix changes, reduced hydration, and progressive degeneration. As a result of the disease process, the NP matrix is destroyed due to increased expression of matrix metalloproteinases and inflammatory factors. This leads to disturbances in the production of NP matrices. This process gradually covers the AF and leads to its microdamage. All these changes are accompanied by disturbances in the function of cells found in the NP, especially progenitor cells. Progenitor cells are defined as tissue-specific stem cells responsible for regenerative processes. They have a limited self-renewal capacity but a high proliferative potential. Progenitor cells maintain homeostasis in the tissue in which they reside and play a major role in regeneration following injury. Notably, intervertebral discs are the largest avascular structures in the human body. This is certainly an additional factor determining the development of degenerative processes. The aim of this report is to analyze metabolic disorders in the degeneration process [1,2].

Nuclear magnetic resonance spectroscopy allows the study of biochemistry and cellular metabolism, not only in the whole organism in vivo (MRS) but also (at higher magnetic field strengths) in vitro (NMR). NMR methodologies have found wide application in biochemistry and medicine. In vitro NMR of cells, body fluids, and tissues is used in medical biochemistry to study pathophysiological processes, and recently, the technique has also been used to verify pathophysiological abnormalities in vivo. NMR is becoming a very important tool in precision medicine and metabolomics. The aim of this report is to present the results of in vitro NMR examinations characteristic of normal (in the T2-weighted image) and degenerate NPs.

In our research on the NP, we used NMR spectroscopy of extracts of hydrophilic and hydrophobic compounds of the tissue. To date, the NP has been tested in MAS NMR, but NP extracts have not been tested. There are no comparative data on NP extracts, hence the idea to determine if and what differences exist in aqueous and chloroform extracts in control and degenerate NPs. In addition, the identification of compounds that distinguish degenerate NPs from control may allow the in vivo spectroscopic measurements to be modified so that these compounds are visible. This will allow the translation from NMR to MRS disc studies and precise determination of degenerate disc localization.

## 2. Results

**Figure 1:**
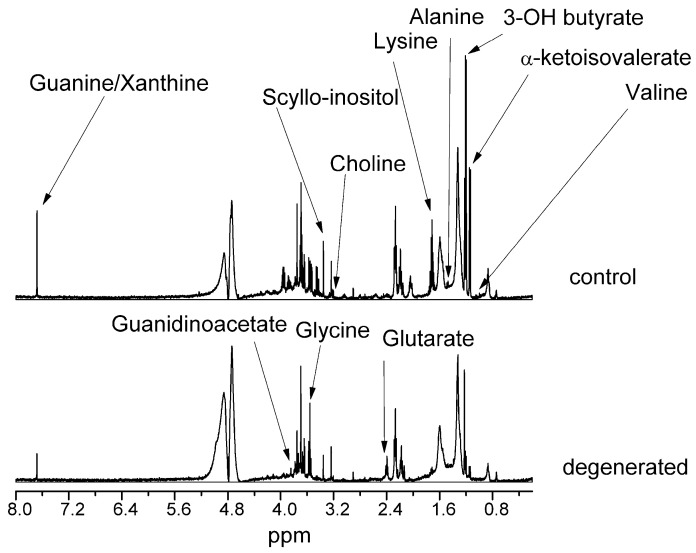
NMR spectra of hydrophilic compounds of NP.

**Figure 2:**
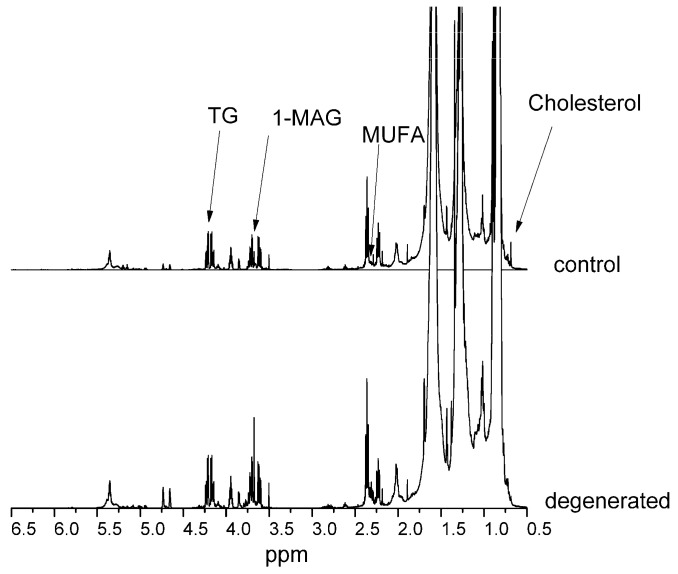
NMR spectra of hydrophobic compounds of control and dehydrated NP: 1-MAG—monoacyloglycerol; TG—triglycerides, MUFA—monounsaturated fatty acid.

Figure 1 and Figure 2 present NMR spectra for control and diseased NPs of hydrophilic and hydrophobic compounds, respectively. Spectra were scaled to a 1 mM TSP internal standard and a CHCl_3_ signal for hydrophilic and hydrophobic compounds, respectively. Spectra were also normalized to the milligram of wet weight of the sample.

In Table 1 and Table 2, we present changes in selected compound signal intensities of the degenerated NP compared to the control NP.

The most changed compounds were valine, guanine/xanthine, 3-hydroxybutyric acid, α-ketoisovaleric acid, glycine, guanidinoacetate, scyllo-inositol, lysine, alanine, and glutaric acid. In the lipid fraction, almost all compounds increased their levels in dehydrated NP, except for free cholesterol, squalene, and fatty acids in all saturated polyunsaturated (PUFA) and monounsaturated (MUFA) forms. There was also a 65% decrease in the unassigned compound signal.

**Figure 3:**
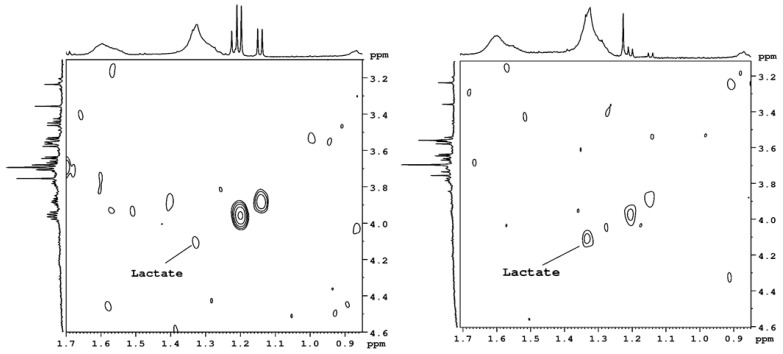
The 2D COSY NMR spectra of the region with the lactate cross-peak. Left-hand—control, right-hand—dehydrated disks.

Figure 3 presents the 2D spectra measured in the homonuclear COSY sequence. The lactic acid cross-peak is more visible in the dehydrated NP than in the control NP. However, in the 1D NMR spectra, the level of lactic acid is small in the control and degenerated NP; therefore, it could not be measured with high accuracy.

## 3. Discussion

Disc degeneration can result from an imbalance between anabolic and catabolic processes or from a loss of balance in the state of metabolism that is maintained in a normal disc. The NMR spectroscopy method is sensitive enough to distinguish between control and diseased intervertebral discs based on metabolite studies. The fact that the study of both discs was performed in one patient allowed us to eliminate the influence of other factors, such as demographic, genetic, or environmental factors. In vitro spectroscopy, due to the high resolution of the spectrum, allowed us to identify compounds that indicate pathology. NMR spectroscopy can also be used in vivo, but the spectral resolution is much lower. On the basis of in vitro tests, it is possible to identify some of the indicated compounds in in vivo tests, e.g., glycine, by appropriately modifying the measurement sequences.

The results of our research collected in Table 3 indicate disturbances in the biosynthesis and metabolism of cholesterol, the biosynthesis and degradation of various FA groups, ketone bodies, or lysine, and the metabolism of glycerophospholipids, purines, glycine, inositol, galactose, alanine, glutamate, and pyruvate in the biosynthesis of valine and isoleucine, leucine. All these disorders indicate pathomechanisms related to oxidative stress, energy, and neurotransmission disturbances and disturbances in the structure and functioning of cell membranes, inflammation, or chronic pain generators.

Mitochondria are a major site of reactive oxygen species (ROS) generation. Mitochondria-dependent ROS production has been reported in various disc cells derived from different species, including human NP and AF cells [3]. Multiple studies have proven that extracellular uric acid at physiological concentrations is a strong antioxidant that can reduce the negative effects of oxidative stress by scavenging approximately 60% of the total-body ROS [4]. Uric acid (UA) is the end product of purine metabolism. In our study, we could not measure UA because it is released outside of the cells, but we could measure earlier products from the purine metabolism cycle—guanine or xanthine. The levels of those compounds drastically decreased in degenerated discs to 30% of normal levels. IVD cells can generate ROS, and ROS overexpression has also been observed in degenerated IVDs. ROS are important mediators in the signal network of IVD cells. They regulate the matrix metabolism, pro-inflammatory phenotype, apoptosis, autophagy, and aging of IVD cells. The oxidative stress involved in ROS not only exacerbates extracellular matrix degradation but also promotes a reduction in the number of living cells in the IVD. When the serum UA level is lower, the antioxidant effect provided by UA is weakened, which may accelerate the progression of intervertebral disc degeneration [5].

Squalene is a cholesterol precursor. In our research, we observed an increase in squalene concentration and a decrease in cholesterol in a damaged disc, which may indicate a defect in the enzymes mediating the conversion of squalene to cholesterol. Due to the pathway from squalene to cholesterol being multistage (KEGG metabolic pathways—Kyoto Encyclopedia of Genes and Genomes—databases), we cannot indicate the disturbance point because we did not see intermediate products due to low concentrations. Increased concentrations of monoacylglycerol (1-MAG), diglycerides, and triglycerides (TG) can lead to increased synthesis and subsequent degradation of various FA groups. Disturbances in FA degradation (KEGG) are also indicated by a 7-fold increased level of glutaric acid in a damaged disc. The accumulation of glutaric acid occurs in the case of glutaric acidosis type I (GA I; OMIM # 231670), which is a disorder of the systemic and brain metabolism of organic acids caused by biallelic variants of glutaryl-CoA dehydrogenase (GCDH), which encodes the mitochondrial, flavin-dependent GCDH that mediates the degradation of lysine, hydroxylysine, and tryptophan [6]. A similarly high level of glutarate is found in type II glutaric acidosis (GA II) or multiple acyl-CoA dehydrogenase deficiency (MADD, OMIM # 231680), which is an inherited autosomal recessive disease affecting the metabolism of fatty acids, amino acids, and choline. In our research, with a high level of glutaric acid, we observed decreases in lysine (by approximately 90%) and choline (by approximately 50%). It has been observed that some people with GA I develop serious complications of the musculoskeletal system. In our research method, we were unable to identify a specific defective enzyme causing disturbances in the metabolism of lysine, choline, or FA, but perhaps the mechanism is similar to that of GA I and II [6,7].

Inflammation is another mechanism that can damage the intervertebral disc. In our research, an indication that this may be the case is increased levels of FA, MUFA, and PUFA. These compounds may be precursors of pro-inflammatory factors in NP cells. In NS cells, there is a balance between pro-inflammatory and anti-inflammatory factors that can be produced biochemically from arachidonic acid and PUFAs. If this state of equilibrium is disturbed, there may be an overproduction of inflammatory factors leading to damage to the intervertebral disc. The explanation of all details of biochemical pathways is in the publication of Das [8].

A tissue or organ can generate pain only if it is innervated. In a healthy disc, nerve endings can be found only in the periphery of the outer annulus fibrosus (AF) and throughout the end plate with varying densities: no nerves are normally present in the inner AF or NP. These nerves are mainly nociceptive, responding to neurotrophins, such as nerve growth factor, and are implicated in disc pain transmission. However, in highly degenerated discs, these nerves can penetrate into the NP. All the tissues that surround the spinal motion segment are innervated and thus have the potential to cause pain [1]. The increased level of glycine in the degenerate disc we observed is particularly important. The glycine receptor (GlyR) is the receptor for the neurotransmitter of the amino acid glycine. GlyR is an ionotropic receptor that acts through a chloride current. It is one of the most widespread inhibitory receptors in the central nervous system and plays an important role in a variety of physiological processes, especially mediating inhibitory neurotransmission in the spinal cord and brainstem. However, the GlyR subtypes contribute differently to nociception and chronic pain. The present evidence strongly suggests that post-translational modifications of synaptic α1β and α3β GlyR subunits are involved in spinal glycinergic disinhibition and chronic inflammatory pain [9]. However, the roles of the α2 and β subunits remain largely unknown. A variety of molecular mechanisms suggest the highly dynamic plasticity of GlyR and other proteins in the glycinergic system. Signal pathways that control GlyR phosphorylation appear to be critical for the fine-tuning of inhibitory synaptic force. The data available thus far suggest that GlyR phosphorylation acts as a molecular switch regulating cell membrane expression, synaptic localization, and ion channel function. Whether these mechanisms work in parallel or independently at glycinergic synapses affected by pain signals remains to be determined. Despite the increasing number of recent reports, it should be noted that characterization of the roles of GlyR subtypes in nociception and chronic pain is far from complete, as many open questions in this area have yet to be resolved. A significant concern is the importance of tonic glycinergic and presynaptic GlyR inhibition in pain control. Another question is whether the supraspinal subtypes of GlyR or GlyR expressed in non-neuronal cells also participate in pain regulation, which is almost completely unexplored. Similarly, the expression and potential importance of GlyR on major afferents remain controversial. Finally, the role of GlyR-targeting autoantibodies in pain control in humans is largely unknown. The identification and characterization of novel protein kinases, protein‒protein interactions, and post-translational modifications involved in GlyR regulation could open exciting new avenues in pain research [9,10,11].

In cell metabolism, intracellular oxygen levels are directly connected with changes in gene expression through the regulation of hypoxia-inducible factor (HIF) and its degradation by the von Hippel–Lindau axis. In the IVD, including the NP, these processes also occur, and proper HIF function is necessary for IVD integrity preservation. Dysregulation of HIF signaling is considered a trigger for degenerative cascade processes, and hypoxia-inducible factor 1α (HIF1α) is indicated as the guardian of hypoxic cells. In NP cells, HIF1α is regulated by both oxygen-dependent and oxygen-independent mechanisms involving prolyl hydroxylase domain-containing proteins (PHDs) and circadian rhythm. These cells have functional mitochondria that undergo HIF-dependent mitophagy and fragmentation. By adjusting cellular metabolism to living within low oxygen levels, the functioning of HIF1α maintains constant energy production levels, intracellular pH, and redox homeostasis and indirectly controls critical cellular processes, such as matrix biosynthesis. HIF1α influences glycolytic flux, in part, by maintaining H+/lactate efflux from NP cells at the final stage of glycolysis. In addition, it controls intracellular pH levels. High lactate levels act as a hypoxia mimetic factor by initiating the biosynthesis of TCA cycle intermediates. Studies in NP cells show a dynamic relationship between metabolic flux and HIF1α activity that is mediated by intracellular lactate levels, where intracellular lactate accumulation increases HIF1α activity [12]. Our research indicates that the mechanism associated with HIV family proteins in the damaged intervertebral disc is disturbed. This is evidenced by lowered levels of lactate and ketone bodies.

Wang et al. [13] proposed a new model of disc metabolism: lactate-dependent metabolic symbiosis. They proposed a working model of disc lactate-dependent metabolic symbiosis whereby hypoxic NP cells anaerobically convert glucose into lactate, which is then secreted and imported into neighboring cells of the more oxygenated AF tissue to be metabolized via oxidative phosphorylation. Their data suggest that lactate has been overlooked as an important biofuel in disc metabolism. Importantly, their findings also indicate the existence of lactate-dependent metabolic symbiosis between the NP and AF of the IVD as a metabolic adaptation to efficiently recycle lactate. Such metabolic adaptation would not only prevent the accumulation of lactate as a waste product but also simultaneously generate the much-needed energy as well as precursors for biosynthesis to cells residing within the nutrient-poor disc environment. One important implication of their model is that compromised or disrupted lactate symbiosis could contribute to metabolic disturbance and intervertebral disc degeneration. In our studies, the lactate level was decreased to 60% in degenerated discs compared to control discs. This may support the theory described by Wang et al. [13].

Reduced disc height associated with disc degeneration can lead to facet overloading and subsequent facet degeneration and pain, which can be exacerbated by increased compression [14]. Although discography has been used widely to localize painful discs and is regarded as the gold standard in the diagnosis of discogenic pain, this procedure is invasive and may even enhance the progression of disc degeneration itself. Unfortunately, none of the standard clinical tests can differentiate discogenic from nondiscogenic pain. For the past two decades, magnetic resonance imaging (MRI) has been the gold standard in assessing disc degeneration severity. In the T2-weighted MRI sequences, water content is correlated positively with signal intensity; therefore, the healthy NP is bright on T2 MRI because it contains a large amount of proteoglycan (PG) that attracts water. In disc degeneration, PG degradation results in a secondary reduction in water content, and consequently, the NP signal darkens on T2 MRI. Various novel MRI approaches have been introduced to detect subtle chemical changes in the disc matrix that may have important clinical value (e.g., T1rho, CEST, UTE, and sodium CSI). However, indications for use are currently not agreed on because prospective studies that validate the utility of these techniques, both in terms of LBP diagnosis and improved clinical outcomes, have not been extensively performed [14].

## 4. Materials and Methods

### 4.1. Patient

**Figure 4:**
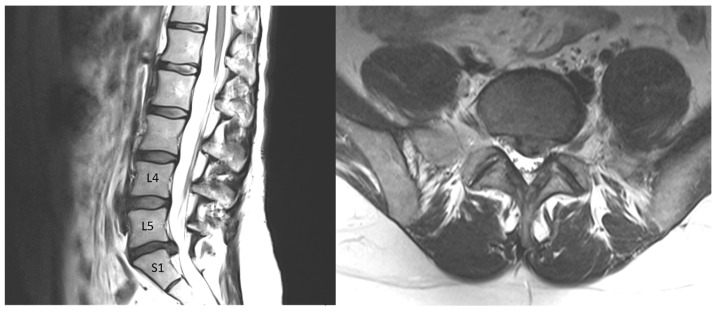
T2-weighted images in sagittal and axial projections. At the L4/5 level, control disc, and at the L5/S1 level, visible left-sided protrusion of dehydrated disc.

A 36-year-old female was admitted to the hospital because of sciatica pain lasting 3 months in the right leg. Routine clinical tests, such as blood morphology and sodium, potassium, chloride, creatinine, and glucose levels, as well as tests of the coagulation system and autoimmunology and rheumatology tests, were performed on the patient before surgery and were within the normal range for age and gender. The symptoms did not subside after conservative treatment. MRI (Figure 4) revealed prolapse at the L5/S1 level of the spine in a degenerated disc (grade IV on the Pfirrmann scale—an inhomogeneous hypointense dark gray disc with significant disc height loss). Minimally invasive discectomy via microscopy was performed. Because the disc tissue had atypical, soft consistency, an additional intraoperative X-ray was performed, and it was noted that the discoidectomy was mistakenly performed at the L4/5 level (grade II on the Pfirrmann scale—an inhomogeneous disc with normal disc height and a clear difference between the nucleus and annulus). An error was noticed, and fenestration with the removal of the disc at the level of L5/S1 from the right side was performed. Careful hemostasis prevented contamination of the tissue with blood. After the procedure, the patient was informed about the course of the procedure and gave her written consent for the examination of the collected tissue from both levels. Recently, the patient has felt well. The disc at level L5/S1 was assigned as a surgical disc and at level L4/5 as a nonsurgical disc.

### 4.2. NMR Spectroscopy

During surgery, the material was put into two marked sterile containers without any fluid, and after the treatment, it was placed in a freezer at −20 °C. Before NMR spectra measurements (a few days later), the material was thawed and washed out with 0.9% NaCl to remove any blood contamination and then dried with tissue paper. The collected NP fragments were weighed for subsequent standardization of the obtained results. Hydrophilic and hydrophobic compounds were extracted from the NP of the intervertebral disc. For the study, two NP samples were used: control (nonsurgical) and dehydrated (surgical). Extraction was performed using the modified Bligh and Dyer method described by Toczylowska et al. [15] with an additional slight modification: the NP was fragmented, and each step of the extraction procedure was extended to 30 min. Three-trimethylsilyl propionic acid (TSP) at a final concentration of 1 mM was used as an internal reference for normalization of all spectra and quantitative statistical analysis. Spectra were measured using proton 1D one pulse with and without presaturation for hydrophilic and hydrophobic compounds, respectively. We also measured 2D spectra using a homonuclear proton COSY pulse sequence. All NMR spectra were acquired at 25 °C using an Avance III HD 500 MHz (Bruker, Germany) spectrometer. For the 1D pulse sequence, 12.5 repetition times and 512 transients were used. For chloroform extracts, one pulse sequence was used with a 5 s repetition time and 128 transients. The spectra were qualitatively analyzed to find differences between healthy and diseased discs.

## 5. Conclusions

The NMR method allowed us to identify compounds, the amount of which in the degenerate disc changed compared to the healthy disc. This study identified compounds that we should find in MRS disc testing in patients. Distinguishing a degenerated disc from a healthy disc using MRS is noninvasive and devoid of subjective errors, which cannot be excluded in the case of X-ray descriptions. We suppose that this method based on assessing changes at the biochemical level will allow for an earlier diagnosis of the source of pain before the morphological changes of the disc will be visible in radiological examinations. Additionally, this could allow for the identification of the pain-generating disc and local administration of new drugs, e.g., glycine receptor modulators.

## Figures and Tables

**Table 1:** Hydrophilic compound percent changes between degenerated and control NP.

Hydrophilic Compounds	Degenerated NP vs. Control (%)
Guanine/Xanthine	41
Galactose	95
Lactic acid	80
Guanidinoacetate	149
Glycerol	141
Glycine	481
Oxalacetic acid	87
Scyllo-inositol	63
Glycerophosphocholine	125
Choline	72
Dimethylglycine	130
Glutaric acid	979
2-oxoadipic acid	105
Acetoacetic acid	111
Lysine	11
Lipids 1 ^1^/2-oxoadipic acid/glutaric acid	111
Alanine	64
Lipids 2/lactic acid	111
3-Hydroxybutyric acid	11
α-Ketoisovaleric acid	13
Valine	0
Lipids 3	83
Lipids 4	148

^1^ Different lipid macrocompounds (including LDL, HDL, and VLDL) are indicated with numbers 1 to 4.

**Table 2:** Hydrophobic compound or functional group percent changes between degenerated and control NP.

Hydrophobic Compounds/Functional Groups	Degenerated NP vs. Control (%)
MUFAs ^1^ (1) ^2^	134
Triglyceride, PUFAs ^3^, and MUFAs	199
Unassigned	46
Squalene	239
Triglyceride	184
1,3-Diglyceride	192
1-Monoglyceride	187
PUFAs (2)	156
PUFAs (3)	162
Saturated FAs ^4^, PUFAs, and MUFAs (1)	640
1,3-Diglyceride, 1-monoglyceride, and FAs	142
MUFAs/squalene	228
PUFAs (4)	147
Saturated FAs, PUFAs, and MUFAs (2)	351
Saturated FAs and PUFAs (1)	203
Saturated FAs and PUFAs (2)	210
Saturated FAs, PUFAs, and MUFAs (3)	236
Saturated FAs	383
PUFAs (5)	225
Free cholesterol	29

^1^ MUFAs—monounsaturated fatty acids; ^2^ (n)—different fatty acid compositions; ^3^ PUFAs—polyunsaturated fatty acids; ^4^ FAs—fatty acids.

**Table 3:** Functions and metabolic pathways of metabolites and directions of changes found in degenerated NP compared to the control are presented.

Metabolites	Metabolic Pathway	Most Likely Pathomechanism	Degenerated NP vs. Control
Squalene	Cholesterol biosynthesis	Energy and neurotransmission disturbances	↑
Cholesterol	Cholesterol metabolism	Energy and neurotransmission disturbances	↓
FA, MUFA, and PUFA	FA biosynthesis	Energy production disturbances, cell membrane function disturbances, and inflammation	↑
1-MAG, DAG, TG	Glycerophospholipid metabolism	Cell membrane function disturbances	↑
Guanine/xanthine Glycine Choline	Purine metabolism Glycine, serine and threonine metabolism, Glycerophospholipid metabolism	Oxidative stress and neurotransmission disturbances, pain generators	↓ ↑ ↓
Glutaric acid Lysine	FA biosynthesis/ degradation, Lysine biosynthesis/ degradation	Energy production disturbances, cell membrane function disturbances	↑ ↓
Lactic acid Alanine Oxalacetic acid	Alanine, glutamate and pyruvate metabolism	Neurotransmission disturbances, oxidative stress	↓ ↓ ↓
3-Hydroxybutyric acid	Synthesis and degradation of ketone bodies	Energy production disturbances	↓
Scyllo-inositol	Inositol metabolism	Energy production disturbances	↓
α-Ketoisovaleric acid Valine	Valine, leucine, and isoleucine biosynthesis	Neurotransmission disturbances, oxidative stress	↓ ↓
Galactose	Galactose metabolism	Energy production disturbances	↓

↑↓—values increased or decreased compared to the control group.

## Data Availability

The data presented in this study are available on request from the corresponding author.
